# Cultural keystone species revisited: are we asking the right questions?

**DOI:** 10.1186/s13002-020-00422-z

**Published:** 2020-11-11

**Authors:** Michael A. Coe, Orou G. Gaoue

**Affiliations:** 1 Department of Botany, University of Hawai‘i at Mānoa, Honolulu, HI 96822 USA; 2 Department of Ecology and Evolutionary Biology, University of Tennessee, Knoxville, TN 37996 USA; 3 Faculty of Agronomy, University of Parakou, Parakou, Benin; 4 Department of Geography, Environmental Management and Energy Studies, University of Johannesburg, APK Campus, Johannesburg, South Africa

**Keywords:** Theory in ethnobotany, Cultural importance indices, Biocultural conservation, Ethnobiology, Conservation biology

## Abstract

The cultural keystone species theory predicts plant species that are culturally important, play a role in resource acquisition, fulfil a psycho-socio-cultural function within a given culture, have high use-value, have an associated naming and terminology in a native language, and a high level of species irreplaceability qualify for cultural keystone species designation. This theory was proposed as a framework for understanding relationships between human societies and species that are integral to their culture. A greater understanding of the dynamic roles of cultural keystones in both ecosystem processes and cultural societies is a foundation for facilitating biocultural conservation. Given such important direct conservation implications of the cultural keystone species theory, we reviewed the use of this theoretical framework across the literature to identify new directions for research. Most studies often emphasized the role of cultural keystones species in human societies but failed to provide a robust and reproducible measure of cultural keystone species status or direct test of the predictions of the theory and underemphasized their potential roles in ecosystem processes. To date, no studies that mentioned cultural keystone species tested the predictions of the theory. Only 4.4% provided a measure for cultural keystone status and 47.4% have cited or applied keystone designation to a given species without providing a reproducible measure for cultural keystone species. Studies that provided a measure for cultural keystone species primarily occurred in North America while few of these studies occurred in Australia and Europe with none occurring in Africa. As such, most cultural keystone species have been designated as such qualitatively based on researcher subjectivity while other studies have designated keystone species with quantitative indices of cultural importance, often incorporating researcher biases or measuring a few of the cultural keystone status predictors rather than all of them, indicating a lack of consensus in identifying cultural keystone species. Thus, we pose the need for a paradigm shift toward the development of serious and systematic approaches for keystone designation.

**Supplementary Information:**

The online version contains supplementary material available at 10.1186/s13002-020-00422-z.

## Introduction

Over two decades ago, ethnobotanists proposed the cultural keystone species concept, an ethnobotanical theoretical framework [1–4] as complementary approach for conservation of social and ecological systems [2, 3, 5–7]. Cultural keystone species are “culturally salient species that shape in a major way the cultural identity of a people, as reflected in the fundamental roles these species have in medicine, materials, diet, and/or spiritual practices” [2] or “species whose existence and symbolic value are essential to the stability of a cultural group over time” [3]. Cultural keystones are often embedded within social and ecological systems where they are thought to play critical roles in maintaining cultural or ecological stability at a local level [2]. Cultural keystones are expected to affect culture, language, and to be irreplaceable therefore, the loss of these species is predicted to have a significant effect on cultural integrity and equilibrium compared to other species that are likely to have little or no effect [2]. In this context, the loss or removal of cultural keystones from their sphere of influence or *ethnosphere* is expected to result in significant cultural community disruptions [2, 3, 8].

Several parallels between cultural and ecological systems have been highlighted in efforts to help define conservation priority and provide a platform for an in-depth understanding of the significant roles cultural keystones can play among social and ecological systems [2]. Garibaldi and Turner [2] proposed a synthesis of the cultural keystone species theory within an ecological context by suggesting “a decline in biological diversity often means a loss of cultural diversity.” This concept has gained momentum where the links between biological, cultural, and linguistic diversity have been identified to help advance our understanding on their potential roles in adding conservation efforts [9–11]. The premise of Garibaldi and Turner’s [2] argument is rooted in the *ecological keystone species* concept which suggests some species are central to ecosystem function where certain species represent *keystones of the biological community* often playing significant roles in maintaining the integrity and longevity of community structure. Thus, it is expected the loss of these species will significantly affect ecosystem function and stability [12, 13]. Further, the ecological keystone species theory was founded on the idea that effective conservation efforts likely depend on understanding the underlying mechanisms by which keystone species play critical roles maintaining stability of their respective ecosystems [14, 15].

While conservation approaches historically focused primarily on ecosystem processes, fundamental components often overlooked are the cultural implications of keystones—which the cultural keystone species theory aims to address. In highlighting relationships between cultural and ecological domains, Garibaldi and Turner [2] posed the idea that certain keystone species are likely to occupy similar functions in both cultural and ecological systems. Thus, suggesting an explicit interconnection between socio-cultural-ecological systems where the functional role cultural keystones are expected to play within the community structure and stability of human societies is analogous to that of the ecological role of keystone species [2]. Given the direct conservation implications, it has been suggested certain keystone species that are both culturally and ecologically important in maintaining socio-ecological dynamics could play important roles in informing management practices coupled with local and ecological knowledge [16].

It is important to mention noted limitations of the ecological keystone theory have long been discussed. There have been persistent calls for action for a functional consensus definition [2, 14, 15, 17, 18] as well as standardized approaches to identify ecological keystones and to quantify the extent to which a given species has an effect on a particular community or ecosystem trait [15, 19, 20]. However, an overall consensus on defining and identifying ecological keystones among ecologists remains to be developed and consistently applied [18]. Regardless, the notion that there is a link between identifying ecological keystones and conservation has become popular in the literature (see for example [14, 15, 21–23]). As such, developing successful conservation and restoration plans likely depends upon understanding the socioecological components such as cultural knowledge [24] and an in-depth understanding of keystone species function [2]. However, the parallels between the critical roles keystone species are predicted to play concomitantly in social and ecological systems have been criticized [5, 7] and a robust test of these predictions has yet to occur. Thus, our understanding of socioecological dynamics of keystone species function and their potential to facilitate biocultural conservation remains limited.

While the overall objective of the cultural keystone species theory is to provide a complementary framework that highlights the mechanisms underlying interrelationships between biological and cultural diversity, discussions surrounding the functional roles of cultural keystones among human societies has been the primary focus in ethnobiological and anthropological research (see for example [2, 3, 25–29]). Researchers have long highlighted the importance of keystones in human societies yet a global synthesis on the effect of keystone species function in terms of the stability of both cultural and ecological domains is lacking as is a standardized and objective approach in identifying keystones.

To identify cultural keystone species Cristancho and Vining [3] as well as Garibaldi and Turner [2] proposed several criteria to determine whether a given species qualifies for keystone designation including (1) intensity, type, and multiplicity of use; (2) abundance; (3) naming and terminology; (4) irreplaceability; (5) use in trade or resource acquisition; (6) psycho-socio-cultural function (e.g., symbolism, knowledge transmission, etc.); and (7) a high level of importance. Though these criteria aim to provide a framework for researchers to clearly identify and measure the extent of which a given species qualifies for cultural keystone designation which, in turn, could be used to provide a direct test of the theory, accurately measuring and defining species cultural keystone status has proven challenging (see for example [30]). Aside from highlighting criteria for cultural keystone designation, Cristancho and Vining [3] did not provide a clear methodology (qualitative or quantitative) to measure cultural keystone status. In contrast, Garibaldi and Turner [2] proposed the use of the index of cultural significance (ICI) to determine whether a given species qualifies for keystone designation. Subsequently, the use of cultural important indices which are expected to measure the *importance of the role a given plant and or animal species plays within a particular culture* [31], have often been used by ethnobiologists to predict cultural keystone status (see for example [2, 25, 32, 33]). These approaches have been criticized [5] as they have yet to provide reliable and reproducible results in identifying cultural keystone species. Consequently, it is unclear whether there is support for the theory or how much progress has been made over the last several decades in testing the theory as well as its use by researchers to determine the keystone status of a given species.

Here, we explore the way in which researchers have been studying cultural keystone species. This review provides a retrospective examination of the cultural keystone species theory while posing a call for action for the development of novel approaches for keystone designation and a direct test of the cultural keystone species theory. We ask, “if studies, rather than testing the relationship between species cultural keystone status and the functional role cultural keystone species are expected to play in maintaining cultural community structure, directly identified cultural keystone species without a robust measure of species cultural keystone status?” We explore how the utilization of the cultural keystone species theory has changed over time and across continents to identify any gaps of knowledge that warrant further considerations. We highlight how far researchers have come in providing a direct test of the cultural keystone species theory, methods used for keystone designation, and encourage a critical examination of how the theory may be used in examining the links between human environmental impacts effecting biological diversity. This review aims to address the following questions including (1) how has the cultural keystone species theory been tested over time and space? (2) How has cultural keystone designation been predicted? (3) What have been the limitations of prior studies that have provided a reproducible measure for cultural keystone species? and (4) What are the future directions for providing a direct test of the theory?

## Methods

We conducted a systematic literature review on cultural keystone species concept, spanning from the application of this theoretical concept in ethnobiology to its current use by researchers from 2003 to 2016. Our publication search was conducted in January 2016 using the key words “cultural keystone species” in PoP (Publish or Perish) software which aims to retrieve and analyze academic citations [34]. Data sources used in the PoP publication search included Crossref, Google Scholar, Google Scholar Profile, PubMed, Scopus, and Web of Science. We found 473 peer-reviewed publications but the search was refined to 409 publications through critical review and exclusion processes discussed below. The literature review as well as the approach used to extract data is described in Table 1.

**Table 1:** Methodology for data collection/exclusion for search conducted in January 2016 using the key words “cultural keystone species” in PoP. Data sources for PoP publication search included Crossref, Google Scholar, Google Scholar Profile, PubMed, Scopus, and Web of Science

Steps	Procedure	Results
Data search	Peer-reviewed paper database search on PoP—Publish or Perish (Harzing, 2007) using key words “cultural keystone species.”	Title, abstract, and keyword information for 473 papers correlated with initial search.
Data review	Screening the title, abstract, keywords, methods, and publication format to exclude those not relevant to study.	409 papers aligned with study/search criteria following screening procedure
Data collection	Downloaded and gained full text access to all that were relevant.	409 downloaded full text with 18 with no access
Data refinement	Key word search papers for cultural keystone species using finder option. Additionally, read publications that specifically focus on/test cultural keystone species criteria defined by Turner and Garibaldi (2004) and Cristancho and Vining (2004).	409 papers were relevant to study criteria.
Data classification	Systematic classification of the 409 relevant papers using 5 defined criteria (randomly cited, test of theory, mention concept, mention species as cultural keystone, review of the theory/concept) integral to gaining insight on the use/application of cultural keystone species theory.	Dataset of 5 defined criteria for each relevant paper
Data analysis	Summarize and analyze data.	Citation of theory over time

The categories for data collection were chosen and defined by the authors to extract data pertaining to this study. These include (1) the *authors mention a species or several species as cultural keystones* in lieu of measuring cultural keystone status, (2) the *authors solely mention the concept of cultural keystone species* rather than discussing a given cultural keystone or measuring keystone status, (3) the authors *review the cultural keystone species concept*, (4) the *authors cite a paper* on or discussing cultural keystones rather than the criteria mentioned above, and (5) the *authors explicitly provide a reproducible measure of cultural keystone status*. Additionally, studies that provided a reproducible measure of cultural keystone species status were classified according to seven cited methods used for cultural keystone species designation including (1) *index of cultural significance* (ICI) adapted from Garibaldi and Turner [2], (2) *use-value index* (UV) adapted from Philips and Gentry [35], (3) *word counts* (WC), (4) *cultural value index* (CV) adapted from Reyes-García et al. [36], (5) *multivariate frequency analysis* (MFA), (6) *cultural significance index* (CSI) following Silva et al. [37], and (7) *participant consensus* (PC). As such, all relevant publications were classified based on these data.

## Results

**Fig. 1:**
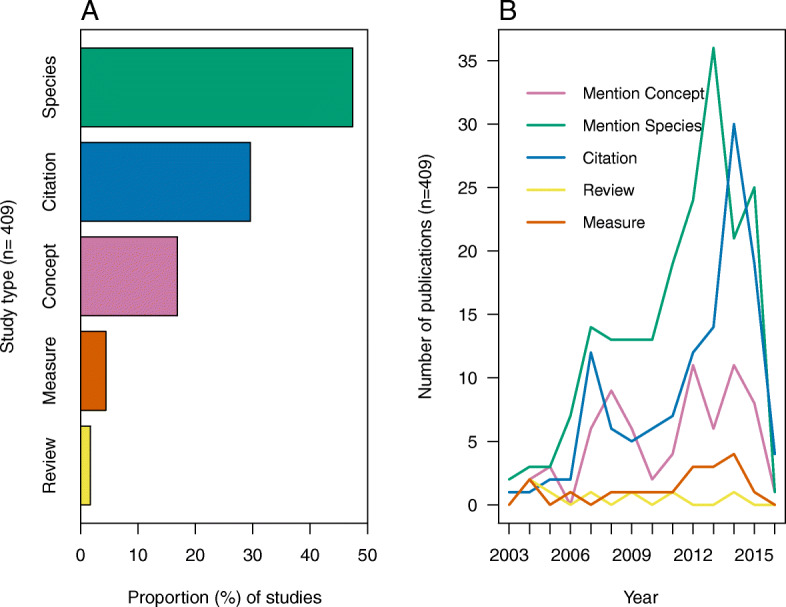
Proportion of studies linked to study type classification (*n* = 409) and the number of publications on cultural keystone species over time (2003–2016) available from Publish or Perish software (*n* = 409). **a** Study type classifications include (1) studies that solely mention the cultural keystone species concept, (2) studies that mention a given species as a cultural keystone species without a direct test or measure of species cultural keystone status, (3) studies that cite a paper on or that discusses the cultural keystone concept, (4) studies that review the cultural keystone species concept, and (5) studies that provide a direct test or measure of species cultural keystone status. **b** Publication types include (1) studies that solely mention the cultural keystone species concept, (2) studies that mention a given species as a cultural keystone species without a direct test or measure of species cultural keystone status, (3) studies that cite a paper on or that discusses the cultural keystone concept, (4) studies that review the cultural keystone species concept, and (5) studies that provide a direct test or measure of species cultural keystone status

A total of 4.4% of the studies that mentioned the words “cultural keystone species” provided a measure of cultural keystone status using one of the seven cited methods, 1.7% reviewed the theory, 29.6% cited a paper on cultural keystones, 16.8% mentioned the cultural keystone concept and 47.4% mentioned a given cultural keystone without explicitly measuring keystone status (Fig. 1a). Further, we found no studies directly tested the predictions of the theory.

Over time, the cultural keystone theory has gained momentum with respect to the study type. Publications that have solely *mentioned a given species* as a cultural keystone, publications that *cited* a given paper on cultural keystones, and publications that *mentioned the cultural keystone concept* have gradually increased over 10 years (2003–2013) (Fig. 1b). However, these study types have been declining since 2013. Publications that *reviewed* the cultural keystone species theory or *provided a reproducible measure for cultural keystone designation* have remained low throughout the study period (Fig. 1b) suggesting most studies have mentioned a given species as a cultural keystone, cited papers on cultural keystone species, or mentioned a cultural keystone species while few studies have provided a measure of species cultural keystone status or have reviewed the cultural keystone species theory (Fig. 1b).

**Fig. 2:**
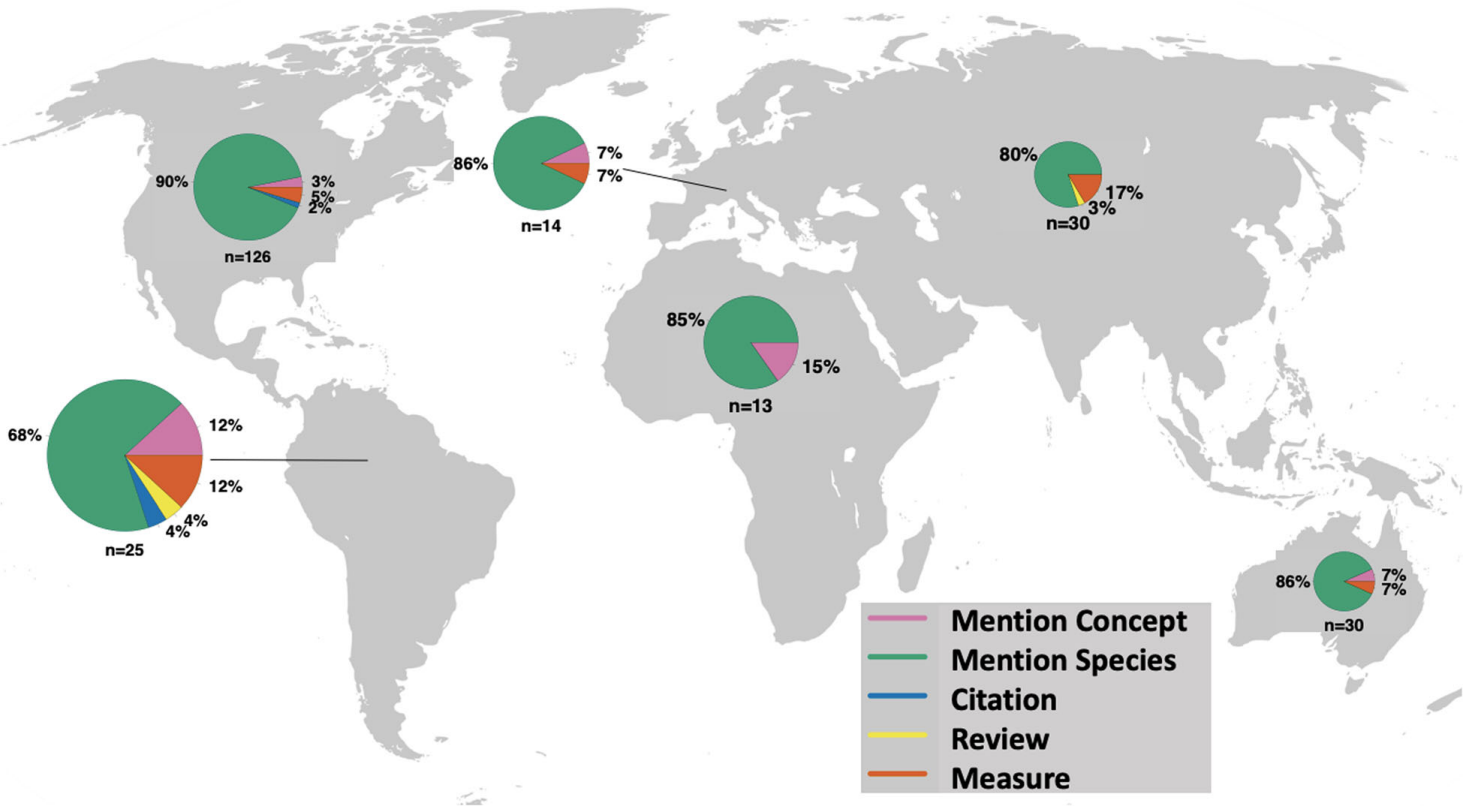
Regional distribution of study classifications linked to cultural keystone species theory (*n* = 238). Study classifications include (1) studies that solely mention the cultural keystone species concept, (2) studies that mention a given species as a cultural keystone species without a direct test or measure of species cultural keystone status, (3) studies that cite a paper on or that discusses the cultural keystone concept, (4) studies that review the cultural keystone species concept, and (5) studies that provide a direct test or measure of species cultural keystone status

**Fig. 3:**
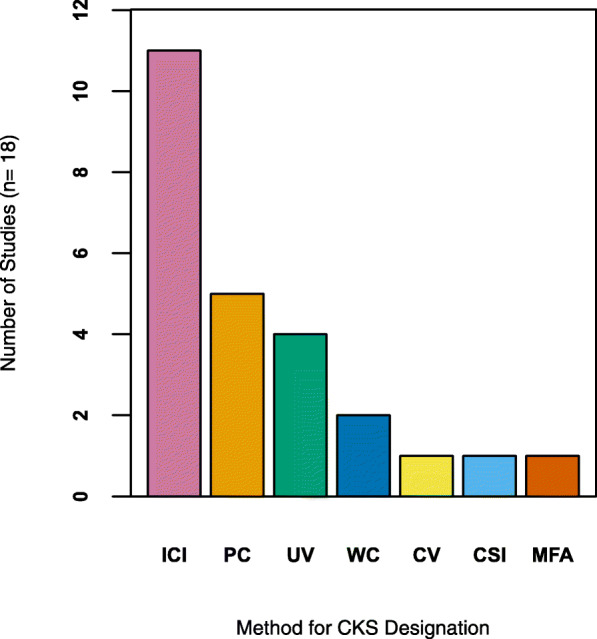
Methods commonly employed for a direct test of cultural keystone species theory (*n* = 18). Methods include the index of cultural significance (ICI), the use-value index (UV), word counts (WC), the cultural value index (CV), multivariate frequency analysis (MFA), the cultural significance index (CSI), and participant consensus (PC)

Our regional differentiation analyses included 238 papers (59%) out of the total number of studies (*N* = 409). Data for studied regions was not available for 171 papers (41%) and subsequent analyses. These papers either mentioned the cultural keystone species concept, cited a species as a cultural keystone, or reviewed the theory without conducting a study on cultural keystone species. Data on studied regions were available for all studies that provided a measure of cultural keystone species status. Globally, most studies to date have mentioned cultural keystone designation (86%, 203 papers total) for a given species without providing a reproducible measure for keystone status (Fig. 2). For example, most studies conducted in Australia or Oceania listed a given species as a cultural keystone species (12.3%, 25 papers) whereas few studies in this area have provided a measure of cultural keystone species status (11%, 2 papers) [38, 39]. Most studies that provided a measure of keystone status occurred in North America (33%, 6 papers). North America also had the greatest number of studies in total (126 papers) with 56% (114 papers) solely mentioning a species as a cultural keystone species, 33% (4 papers) mentioning the cultural keystone species concept [40–43], 66% (2 papers) solely citing a paper on cultural keystone species [44, 45] and no review papers on the cultural keystone species theory (Fig. 3). This suggests, regardless of classification criteria for study type, most studies on cultural keystone species have been conducted in North America which is not surprising considering North America was where the cultural keystone species theory originated. In contrast, the fewest number of studies on cultural keystone species in total (13 papers) occurred in Africa with 5.4% (11 papers) solely mentioning a species as a cultural keystone species, 0.09% (2 papers) that solely mention the cultural keystones species concept (see for example [46, 47]), and no studies that cited, reviewed, or provided a measure of keystone status or tested the cultural keystone species theory suggesting the diversity of studies investigating cultural keystone species on certain continents such as Africa, Australia, and Europe is limited or nonexistent (Fig. 3).

Studies that provided a reproducible measure (*n* = 18 papers) of cultural keystone species status used a variety of methodologies to identify cultural keystone species (Additional file 1: Appendix 1, Table S1; Fig. 3). Out of the seven cited methods (Fig. 3), several have been used concurrently including the use-value index (UV) adapted from Philips and Gentry [35] and the index of cultural significance (ICI) adapted from Garibaldi and Turner [2] or the index of cultural significance (ICI) combined with participant consensus (PC). This was always the case with respect to participant consensus (PC), which was often used (5 papers, 28%) in conjunction with cultural importance indices or was a component of a given index [25, 26, 30, 39, 48]. Most authors (61%, 11 papers) cited the use of the index of cultural significance (ICI) to infer cultural keystone status [2, 25, 30, 32, 33, 49–53]. The use-value index (UV) was used for keystone designation in 22% (4 papers) of studies that tested the cultural keystone theory [26, 32, 49, 54]. Word counts (11%, 2 papers) were either used by themselves [55] or in addition to the proportion of participants that mentioned a given species for keystone designation [38]; Fig. 3). Several authors cited other indices of cultural importance including the cultural value index (CV) (5.5%, 1 paper) and the cultural significance index (CSI) (5.5%, 1 paper) to infer cultural keystone status [39, 48]. Finally, one author cited multivariate frequency analysis (5.5%, 1 paper) for keystone designation [29] (Fig. 3).

## Discussion

We have shown how the cultural keystone species theory has been applied on both a temporal and spatial scale. Since Cristancho and Vining’s [3] and Garibaldi and Turner’s [2] elaboration of cultural keystone species concept [4], the theoretical framework has clearly gained momentum over time and been applied across geographic ranges. Although it is expected most studies that provided a measure for cultural keystone species occurred in North America where the idea of using and index to infer cultural keystone designation originated [2], it is surprising to note the lack thereof or a limited measure of cultural keystone species status in continents such as Africa, South America, and Europe—especially given that certain culturally important plant species in regions such as these have been shown to be deeply rooted in cultural community structure and local livelihoods [26, 56, 57]. This indicates over time, these areas and moreover the cultures linked to them, are largely understudied with respect to cultural keystones.

Surprisingly, no studies provided a direct test of the cultural keystones species theory while few provided a measure of cultural keystone species status. Out of the total number of studies, less than 5% have provided a reproducible measure of cultural keystone species status (Additional file 1: Table S1; Fig. 1). As such, most cultural keystone species designations have been applied without a robust measure of species cultural keystone status where the majority of studies either mentioned the cultural keystone species concept or species related to it (approximately 50%) (Fig. 3). This supports our initial prediction in that few studies have provided a reproducible measure of cultural keystone species status. Given our findings and the conservation implications of the cultural keystone species theory we ask, “why are reproducible measures for cultural keystone species rarely used?”, “why is a direct test of the theory lacking?”, “why is it important to test the theory?”, and “what does testing the theory offer in support of cultural keystone species designation?”

### Is there a robust test of the cultural keystone species theory?

The relative trend in identifying cultural keystone species has consisted of a variety of methods while no studies to date have provided a direct test of the theory. Although it may be expected the index of cultural significance proposed by Garibaldi and Turner [2] would serve as an exclusive approach to designate keystone status based on the reproducibility, our results demonstrate the lack of consistent approaches employed for measuring keystone status (Fig. 3). For example, numerous studies did not explicitly identify cultural keystones based on measuring all the proposed indicators of cultural keystone status (i.e., use value, abundance, naming and terminology, irreplaceability, use in trade or resource acquisition, psycho-socio-cultural function and a high level of importance) highlighted by Cristancho and Vining [3] and Garibaldi and Turner [2]. Instead, researchers often focused on measuring one to several keystone criteria (see for example [29, 38, 56]) to infer keystone designation rather than all of them. As we have shown, most designated keystones were defined as such primarily based on researcher judgement or inference without a reproducible measure of cultural keystone species status or a direct test of the theory (see for example [58–63]; see also Fig. 1). This is likely because Garibaldi and Turner [2] did not highlight explicitly which indicators should be used to directly measure the state of the seven criteria for cultural keystone designation, how such indicators should be scored objectively, and a quantitative threshold to determine the level of importance for cultural keystone species designation. Without a direct test of the theory that is reproducible by the consistent use of standardized approaches to measure species cultural keystone status, we lack an in-depth understanding on whether there is support for the cultural keystone species theory and the potential roles cultural keystone species may play in defining conservation priority. Therefore, we suggest the use of consistent and reproducible methods to identify cultural keystones and to test the cultural keystone species theory is critical to help advance our understanding of the practical application of cultural keystone species designation in conservation biology, to synthesize global patterns or trends linked to cultural keystone species and to provide opportunities to refine the theory. This brings into question, “what methods are most used and appropriate for cultural keystone species designation?”

### Qualitative approaches to understanding cultural keystone species

There is no doubt that qualitative approaches provided in-depth understanding of complex systems on a local scale [64]. Although several researchers defined a given species as a cultural keystone species primarily based on qualitative data alone to infer keystone status (see for example [3, 38, 56]), it is unclear what these approaches may yield in the long-term with respect to reproducibility and global syntheses and application in conservation biology. Given the broad application of methods employed to investigate the cultural keystone species theory, it is important to consider the overarching objectives of a given study as they may not be focused on the application of the cultural keystone species theory for conservation approaches or global inferences. As such, it is possible that researchers’ objectives are to conduct a general ethnobotanical study rather than testing the central predictions of the cultural keystone species theory where a designated cultural keystone species is based on a posteriori in lieu of a direct measure of cultural keystone species status. In these cases, qualitative assessments of a species’ importance to a given culture are likely adequate to yield informative results on a local scale where quantitative assessments of cultural importance are either not needed or too challenging to assess. Although the cultural keystone species theory was proposed primarily as a complementary approach to aiding conservation efforts [2], valid arguments could be made for whether cultural keystone status is best observed at a local level through qualitative methodologies often employed by anthropologists or for whether the theoretical framework could be adequately applied on a broader scale through standardized quantification often employed by interdisciplinary and natural scientists. Regardless of these approaches, it is important for researchers to acknowledge the objectives of a given study and the potential biases of the methods employed. This highlights the fundamental challenges in testing the cultural keystone species theory, determining cultural keystone status of a given species and its application in informing conservation. While discussions surrounding the appropriate use of qualitative and quantitative methods in conservation biology has become widespread, it has often been suggested that interdisciplinary approaches involving complementary frameworks from both social and natural sciences may yield sound results [64, 65].

### Quantitative cultural importance indices as proxy for cultural keystone status

The use of quantitative indices to measure the cultural importance of a given species is widespread in ethnobiology [66–68]. As we have shown, cultural importance indices were most often used to provide a measure of cultural keystone species status. Although the primary aim of these indices is to estimate species *cultural importance* [36, 37, 68–72]*,* several of them were used to predict cultural keystone status ([26, 39, 48]; see also Fig. 3). For example, Garibaldi and Turner [2] were the first to propose a standardized methodology for predicting keystone status through the use of the index of cultural significance. This index including subsequent versions were the most widely used approach to determine if a given species qualifies for keystone designation ([25, 30, 32, 33, 49–53]; see also Fig. 3). Although these approaches have yielded interesting results, a significant limitation of Garibaldi and Turner’s [2] index is the potential for incorporating researcher biases in terms of directly assigning value or scores to the predictors of keystone designation (see [68, 71, 73]). Directly assigning value or weight to the indicators of cultural keystone designation may not accurately account for the emic (view from an individual within a given culture) perspective in terms of cultural keystone species designation. Therefore, cultural keystone designations based solely on this index may be misleading. This illustrates the importance of considering the reliability of the data collected given the methods employed. Further, it is also important to consider the appropriate use of a given index based on the questions addressed or hypotheses being tested [74].

Several authors have acknowledged the limitations of Garibaldi and Turner’s index and modified it to account for participant consensus [25, 30] or used it in conjunction with the use value index adapted from Philips and Gentry [35] in attempts to maximize objectivity [32, 49]. Whereas other authors have employed preferential ranking as well as the cultural value index [48] adapted from Reyes-García et al. [36], the cultural significance index [39] following Silva et al. [37], or the use value index by itself [26, 54] to predict species cultural keystone status. These approaches yielded mixed results (see for example [30]) in identifying cultural keystone species. Therefore, the use of cultural importance indices alone may not be sufficient to measure species cultural keystone status [25]. As such, there is no consensus among researchers on robust quantitative approaches to predict cultural keystone status. Given cultural importance indices were originally defined to quantify species cultural values, it is critical to consider their intended use rather than a panacea used to infer cultural keystone status. Cristancho and Vining [3] included a high level of cultural importance in their proposed keystone designation criteria. However, most cultural importance indices are correlated among each other and do not clearly measure species cultural keystone status [75].

### An objective and robust measure of species cultural keystone status

In light of the challenges in providing a direct and robust measure of species cultural keystone status, we offer a simple and objective way to identify cultural keystone species based on perspectives described elsewhere [75]. It is important to first accurately collect data to estimate variables that measure the criteria known to define cultural keystone species. These criteria include species use values (i.e., intensity, type, and multiplicity of use), species role for resource acquisition, species psycho-socio-cultural value, species ethnotaxonomic diversity, species irreplaceability or level of unique position, and high level of importance. Because some of these criteria are cultural constructs, they are estimated using set of variables to ensure objectivity. A principal component analysis (PCA) [76] of these predictors of cultural keystone status (feasible in *R* [77] using the LABDSV package [78]) will generate principal components which represent unique combination of these predictors. Each principal component provides a score for each species. The first set of principal components which together explain more than 50% of the variance of data can be selected for subsequent analysis. A cultural keystone status score can then be estimated for each species by multiplying the scores of these principal components (see [75]).

It is clear prior approaches to estimate the cultural keystone status of a given species have often failed to objectively account for the proposed criteria for cultural keystone species designation. Though we offer alternative methods, an in-depth understanding of cultural keystone status likely requires long-term periods of fieldwork and critical engagement in the sociocultural practices of a given culture. Finally, it is important to consider if community-based approaches that include preferential ranking or consensus according to the participants’ emic perspective may aid in defining predictor values of a given index thus, providing a reproducible measure of the local perception of species cultural keystone status. As such, the emic perspective of the defining factors for cultural keystone species could be used for quantifying keystone designation and to provide a means to compare these results to those obtained from indices guided by the etic or researcher’s point of view.

### Toward a direct test of the cultural keystone species theory

Though few studies have provided a measure for cultural keystone species status, it is clear there has yet to be a study to date that has provided a direct test of the cultural keystone species theory. By definition, the cultural keystone species theory suggests certain species are so culturally important that their disappearance will significantly affect the cultural integrity of a given community [2] meaning these species are expected to significantly affect cultural frameworks of a given community if they disappear. A first step into testing this theory is to ask “Do cultural keystone species actually exist in all cultural communities?” and “ How will the loss of these cultural keystone species alter community cultural integrity?” Answering these questions requires estimating the cultural keystone scores for species listed by a community of interest and a deep understanding of what constitutes “culture”, what are its components and how to determine if and when a given cultural integrity is altered. As such, it is important that culture and cultural integrity are operationalized and defined by the fundamental components of culture so that the rarity or disappearance of a given high ranking cultural keystone species will be estimated to provide a test of the cultural keystone species theory. The fundamental components of culture [2] are material and non-material elements [79, 80] such as medicine, materials, diet, symbolic values, and spiritual practices [2] of a given cultural community. In defining culture and the elements of culture, it is important to use an emic approach and focus group discussions [66, 81] to determine how the studied community defines their culture.

To test the effect of the loss or extinction of each species on cultural integrity, we suggest conducting simulation techniques [82] with participants. For example, the researcher can present all species previously free-listed by participants. Then by randomly removing and replacing one species at a time, participants will be presented with set of plants lists with one species missing at a time. Following the removal of a given species, participants will be asked “if this species is removed and no longer available to use among the species that are presented, would this change any elements of your culture?” To reduce the potential for researcher bias, all responses are recorded as binary data (i.e., yes/no; 0 or 1) and represent the risk of change in cultural integrity. One way to add precision to this method would be to ask for more details. For example, instead of asking about if the culture will be altered, one can ask if and how each element of culture as defined by the community (e.g., language, symbols, norms, beliefs, rituals, value) will be altered. For each element of culture, binary scores (1/0) or points will be given according to the answer given by participants. In this case, the risk of change in cultural integrity will be obtained by summing the scores across all elements of culture for each species divided by the total number of elements. This approach could also be applied to cultural keystone complexes [5] where a 2 by 2 simulation removal keystone species or a group of species that are used concomitantly is used to evaluate the effect of the loss of these species on the cultural integrity of a given community. At this point the researcher obtains for each species its cultural keystone score obtained using the PCA method and its cultural integrity score measured using simulation interview. A binomial (if binary scores are used) or beta (if the proportion scores are used) regression [83] of these data can be used to test whether species with high cultural keystone scores are likely to have high risk of cultural integrity score. This will provide a direct test of the cultural keystone species theory.

### Call for action

Some authors concluded that the inherent value in the cultural keystone species concept is merely a *process of exploration* rather than the quantification of cultural significance [30], and others have continued to support the idea that it is useful tool for conservation and restoration [84]. Whether researchers employ qualitative, quantitative, or interdisciplinary methodologies for keystone designation it is clear there are inherent limitations, potential biases, as well as advantages in all of these approaches. In light of our results and in efforts to contribute to the ongoing debate, we ask, “if researchers are solely using the cultural keystone designation to suggest the conservation of plants, [2] animals, [38], insects [33], or places [27]? If so, we argue if progress is to be made in identifying cultural keystone species and applying cultural keystone status for effective conservation efforts then, it is critical for researchers to approach the cultural keystone species theory in a serious systematic way—to think critically about how to accurately define and measure cultural keystone designation.

We acknowledge sound conservation efforts informed by cultural keystone designations are likely achieved with a comprehensive understanding of community-based approaches deeply rooted and interwoven within diverse cultural practices coupled with an in-depth knowledge of ecosystem dynamics and sustainable harvest limits [9, 85]. Given challenges faced by human societies and threatened species are constantly in flux, we suggest management practices and cultural keystone designations should be evaluated on a continuous or periodic spectrum as knowledge is gained thus, facilitating flexibility and an evolving understanding of cultural keystone species dynamics and biocultural conservation.

## Supplementary Information

**Additional file 1:** **Table S1.** Publications providing a reproducible measure for cultural keystone species

## Data Availability

The data generated and analyzed during the current study are available in the DRYAD repository, 10.5061/dryad.15dv41nvh.
