# Supplementary material for: Cultural keystone species revisited: are we asking the right questions?
Source: J Ethnobiol Ethnomed. 2020 Nov 11;16:70. doi: 10.1186/s13002-020-00422-z (PMC7657362; doi:10.1186/s13002-020-00422-z)
Supplement: Supplementary file 1 — Additional file 1: Table S1. Publications providing a reproducible measure for cultural keystone species [file 13002_2020_422_MOESM1_ESM.docx]

**Supplementary material: Appendix 1**

Table S1. Publications providing a reproducible measure for cultural keystone species.

|  |
| --- |
| 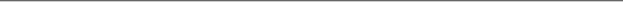 |
| 1. Garibaldi, A., & Turner, N. (2004). Cultural keystone species: implications for ecological conservation and restoration. *Ecology and society*, *9*(3). 2. Wello, Y. E. (2008). Species kunci budaya (cultural keystone species) masyarakat Sumba di sekitar Taman Nasional Manupeu Tanadaru, Nusa Tenggara Timur. *Skripsi. Dep. Konservasi Sumberdaya Hutan dan Ekowisata. Fakultas Kehutanan IPB*. 3. Jackson, R., & Jain, N. (2006). Mountain Cultures, Keystone Species: Exploring the Role of Cultural Keystone Species in Central Asia. Snow Leopard Conservancy, 47 4. Barnes, J. I. (2009). *Cacao: a cultural keystone species among the Kuna of three communities in San Blas, Panama* (Doctoral dissertation, Carleton University). 5. Garibaldi, A., & Straker, J. (2009). Cultural keystone species in oil sands mine reclamation, Fort McKay, Alberta, Canada. British Columbia Mine Reclamation Symposium 2009. *Faculty of Applied Science*. 6. Garine, É. (2007). Quand il n’y a pas d’animal “clef de voûte” dans la zoologie populaire : notes de terrain sur une civilisation agraire du nord du Cameroun (Duupa, Massif de Poli) = When there is no “keystone” species in the folk zoology : *field notes from an agrarian civ (Dounias Ed). IRD Éditions*. 7. Camacho, L. I. C. (2011). Conocimiento etnobotánico, patrones de uso y manejo de plantas útiles en la cuenca del río Cane-Iguaque (Boyacá-Colombia): una aproximación desde los sistemas de uso de la biodiversidad. *Ambiente & Sociedade*, *14*(1), 45-75. 8. Assis, A. L., Hanazaki, N., Reis, M. S., Mattos, A. G., & Peroni, N. (2010). Espécie-chave cultural: indicadores e aplicabilidade em etnoecologia. *Etnoecologia em perspectiva: natureza, cultura e conservação, NUPEEA, Recife, PE, Brazil, pp145–153*. 9. Brandt, R., Zimmermann, H., Hensen, I., Castro, J. C. M., & Rist, S. (2012). Agroforestry species of the Bolivian Andes: an integrated assessment of ecological, economic and socio-cultural plant values. *Agroforestry systems*, *86*(1), 1-16. 10. Butler, A. R., Toh, I., Wagambie, D., Toh, I., & Wagambie, D. (2012). The integration of indigenous knowledge into mine site rehabilitation and closure planning at Ok Tedi, Papua New Guinea. *Mine Closure*, 611–626. 11. Salazar, G. M., Montijo, B. S., Bañuelos, R. S., Alejandra, C., Flores, C., Gamez Duarte, E. A., … Hernández, D. B. (2012). La mariposa de los ténabaris (Rotschildia cinta): cultura, biogeografía y ecología. 12. Uprety, Y., Asselin, H., & Bergeron, Y. (2013). Cultural importance of white pine (Pinus strobus L.) to the Kitcisakik Algonquin community of western Quebec, Canada. *Canadian journal of forest research*, *43*(6), 544-551. 13. Shrestha, S. (2013). Global Localism at the Manaslu Conservation Area in the Eastern Himalaya, Nepal: Integrating Forest Ecological and Ethnobotanical Knowledge for Biodiversity conservation. (Doctoral dissertation, Miami University) 14. Franco, F., Ghani, B. A. A., & Hidayati, S. (2014). Biocultural importance of the Tanying [Koompassia excelsa (Becc.) Taub.] tree for the Berawan of Loagan Bunut, Sarawak, Malaysia. *Indian Journal of Traditional Knowledge*, *13*(1), 63-69. 15. Franco, F. M., Ghani, B. A. A., & Hidayati, S. (2014). Terras (Eusideroxylon zwageri Teijsm. & Binn.), a Cultural Keystone Species of the Berawan People of Sarawak, Malaysia. *Pertanika Journal of Social Sciences & Humanities*, *22*(3). 16. Lefler, B. J. (2014). *Nuwuvi (Southern Paiute) Ecological Knowledge of Piñon-JuniperWoodlands: Implications for Conservation and Sustainable Resource Use in TwoSouthern Nevada Protected Areas* (Doctoral dissertation, Portland State University). 17. Mccarthy, A., Hepburn, C., Scott, N., Schweikert, K., Turner, R., & Moller, H. (2014). Local people see and care most? Severe depletion of inshore fisheries and its consequences for Māori communities in New Zealand. *Aquatic Conservation: Marine and Freshwater Ecosystems*, *24*(3), 369-390. 18. Quave, C. L., & Pieroni, A. (2015). A reservoir of ethnobotanical knowledge informs resilient food security and health strategies in the Balkans. *Nature Plants*, *1*(2), 14021. |
